# Biomarkers and Risk Factors Associated with Apnea in Hospitalized Infants with Acute Respiratory Infection

**DOI:** 10.3390/v18070778

**Published:** 2026-07-15

**Authors:** Julia Dvorkin, Maria Pico, Josefina L. Razzini, Romina Libster, Fernando P. Polack, Mauricio T. Caballero, on behalf of The INFANT Respiratory Network

**Affiliations:** 1 Centro Infant de Medicina Traslacional (CIMeT), Escuela de Bio y Nanotecnologías (EByN), Universidad Nacional de San Martín (UNSAM), General San Martín 1650, Argentina; 2 Consejo Nacional de Investigaciones Científicas y Técnicas (CONICET), Ciudad Autónoma de Buenos Aires 1425, Argentina; 3 Genetics, Vaccines and Pediatric Infectious Diseases Research Group (GENVIP), Instituto de Investigación Sanitaria de Santiago de Compostela (IDIS), University of Santiago de Compostela (USC), 15706 Santiago de Compostela, Galicia, Spain; 4 Fundación INFANT, Ciudad Autónoma de Buenos Aires 1406, Argentina

**Keywords:** apnea, respiratory syncytial virus, prematurity, acute respiratory infection, biomarkers, infants

## Abstract

Apnea is a potentially life-threatening complication of severe acute respiratory infection (SARI) in early infancy; however, its determinants remain incompletely understood. Between 2011 and 2013, we conducted a population-based, cross-sectional, multicenter study in Buenos Aires, Argentina. We examined epidemiological, clinical, virological, and immunological factors associated with apnea in a population-based cohort of 2376 infants younger than five months hospitalized due to SARI. Using hierarchical multivariable logistic regression, we identified preterm birth, cesarean section, previous NICU admission, age under two months, and severe household crowding as independent predictors of apnea. RSV infection was inversely associated with apnea, and IL-5 levels in respiratory secretions were significantly lower among infants with apnea, suggesting a qualitatively different immune response in this group. Taken together, these findings indicate that apnea during SARI in early infancy is primarily driven by biological immaturity and adverse environmental conditions.

## 1. Introduction

Severe acute respiratory infections (SARIs) are a major cause of morbidity and mortality in post-neonatal young infants worldwide [1,2]. A potentially life-threatening complication of SARIs in this age group is apnea, defined as a transient absence of spontaneous ventilation, often accompanied by hypoxemia, pallor, cyanosis, bradycardia, and reduced responsiveness [3,4,5]. Apnea is also one of the most severe complications of respiratory syncytial virus (RSV) infection and other respiratory pathogens, especially in the first three months of life [6]. Notably, apnea or a brief resolved unexplained event (BRUE) may be one of the earliest life-threatening clinical signs in very young infants with RSV infection [6,7,8,9]. For that reason, parents commonly report these episodes even in the absence of respiratory symptoms [8,9]. Without prompt recognition and intervention, apnea can progress to a fatal outcome if gasping or normal breathing is not restored [10].

Apnea secondary to or associated with bronchiolitis in infants is generally managed with supportive and respiratory care, which may include admission to pediatric intensive care units (PICUs), noninvasive ventilation, or mechanical ventilation [3,11]. While prematurity and young age are frequently recognized as risk factors for apnea during SARI, the mechanisms underlying their contribution remain incompletely understood [4]. Therefore, precise identification of infants at high risk for apnea, along with a deeper understanding of the physiopathology underlying this syndrome, is essential for establishing effective preventive interventions in the future.

Predicting apnea in infants with SARI solely based on sociodemographic and clinical risk factors can be challenging. Identifying the pathophysiologic pathways underlying apnea caused by RSV or other respiratory pathogens is crucial for the discovery of potential biomarkers and the development of new preventive strategies against these life-threatening complications. Although limited hypotheses have been proposed regarding the origin of SARI-related apnea, the most widely accepted suggests that it stems from inflammation-induced interleukin-1β (IL-1β) production [12]. This, in turn, triggers the release of prostaglandin E2 (PGE2) in the capillary endothelium of the brainstem, ultimately suppressing the activity of neurons responsible for respiratory control [13].

This study aimed to identify epidemiological, clinical, viral, and immunological factors associated with apnea among hospitalized infants younger than 5 months with SARI. Additionally, we aimed to characterize risk factors associated with apnea among infants with confirmed RSV infection, in order to identify a biologically vulnerable phenotype at highest risk and inform future preventive strategies.

## 2. Methods

### 2.1. Study Population

Between 2011 and 2013, we conducted a population-based, cross-sectional, multicenter study involving 12 pediatric departments of the Southern region of the Metropolitan Area of Buenos Aires (MABA), Argentina, covering a well-defined population of live births without medical insurance. The study was designed to determine the burden and risk factors associated with severe disease and mortality due to SARI in infants under 5 months of age [14]. The present study is a secondary analysis of that cohort, examining the occurrence and risk factors associated with apnea.

Severe acute respiratory infection was defined as an acute respiratory infection with reported or measured fever of ≥38 °C and cough, with onset within the last 10 days, requiring hospitalization [15]. Apnea was defined as an unexplained episode of cessation of breathing for 20 s or longer or a shorter respiratory pause associated with bradycardia, cyanosis, pallor, and/or marked hypotonia [4]. The attending pediatrician diagnosed apnea during SARI, based on parental descriptions of events at home and/or clinical observation in the hospital.

All participating parents or guardians provided written informed consent. The study received approval from the institutional review boards of each participating institution, including Vanderbilt University and the State of Buenos Aires.

### 2.2. Data Collection and Laboratory Procedures

Epidemiological information was obtained from parents or guardians. Clinical data were recorded during the hospital stay until discharge. Risk factors traditionally associated with hospitalization due to SARI and apnea were evaluated. Sociodemographic factors included teenage motherhood (age 19 or younger), low maternal educational level (mothers with incomplete primary education or less), lack of sewage services, household crowding (three or more persons per room) or severe household crowding (five or more persons per room), smoking at home, and precarious housing (built with tin, wood, or mud) [16]. Perinatal and neonatal history included gestational age at birth, prematurity (defined as <37 weeks’ gestation), intrauterine growth restriction (IUGR), and history of previous hospitalizations. Clinical characteristics assessed at the time of hospitalization included sex, age in months, underlying chronic illness (immunodeficiency, congenital heart disease, or neurological disorder), exclusive breastfeeding, oxygen saturation at admission, and weight-for-age Z-score estimated based on local growth charts from the Argentine Society of Pediatrics. Viral etiological diagnosis was performed by obtaining nasal aspirates from all hospitalized infants, which were tested in duplicate for respiratory syncytial virus (RSV), human rhinovirus (hRV), influenza virus (Flu), and human metapneumovirus (hMPV) by real-time polymerase chain reaction (RT-PCR) [14,17]. Viral coinfection was defined as the detection of two or more respiratory viruses during the same hospitalization episode. Nasal aspirates were additionally tested for pro-inflammatory (IL-5, TNF-α, and IFN-γ) and anti-inflammatory cytokines (IL-9 and IL-13) using enzyme-linked immunoassay (ELISA) kits from eBioscience, following the manufacturer’s instructions. Cytokines were selected among inflammatory molecules previously hypothesized to influence the development of apnea (TNF-α) [4] and adaptive T-helper (Th) cytokines associated with the severity of RSV bronchiolitis (IL-5, IL-9, IL-13, and IFN-γ) [18,19,20]. IL-1β was additionally measured but detected in only 24 samples and was therefore not analyzed further. Cytokine measurements were performed on a subset of samples due to limited reagent availability.

### 2.3. Statistical Analysis

Continuous variables were summarized as medians and interquartile ranges, and categorical variables as frequencies and percentages. Univariable comparisons of epidemiological, clinical, viral, and immune variables between groups were performed using Pearson’s χ^2^ test or Fisher’s exact test for categorical variables, and the Mann–Whitney U test for continuous variables, as appropriate. A two-sided *p*-value < 0.05 was considered statistically significant. Variables associated with the outcome in univariable analyses and considered clinically relevant were included in multivariable logistic regression models. Adjusted odds ratios (aORs) and 95% confidence intervals (CIs) were estimated using generalized linear models with a binomial distribution and logit link. A hierarchical modeling strategy was used, following the conceptual-framework approach previously described [14,21]. Variables were entered sequentially in three levels ordered by their conceptual proximity to the outcome—their position in the causal pathway leading to apnea rather than the strength of their association: sociodemographic (first, most distal), biological (second), and clinical/biomarker (third, most proximal). At each level, odds ratios were adjusted for the variables in that level and in the preceding levels; more proximal variables were treated as potential mediators and not used for adjustment. For secondary analyses restricted to RSV-positive infants and critically ill infants, univariable logistic regression models were used, given the limited number of events available for adjustment. All statistical analyses were performed using RStudio software (R version 4.2.2).

## 3. Results

### 3.1. Patient Characteristics Associated with Apnea

During the winter seasons of 2011–2013, a total of 2376 infants under 5 months of age hospitalized with SARI were enrolled. Among them, 77 apnea events were documented, corresponding to a cumulative incidence of 3.24 episodes per 100 hospitalized infants in this age group.

Infectious viral pathogens were measured in all the participants, with a total positive rate of 73.23% (1740/2376). The most common pathogen detected was RSV (54.21%), followed by hRV (10.14%), HMPV (4.88%), and Flu (1.73%). We found respiratory virus co-infections in 6.69% (159/2376) of participating infants. The co-infection rate among hospitalized infants with apnea-associated SARI was 7.79% (6/77).

To identify factors associated with apnea, we first examined the influence of sociodemographic and environmental variables (Table 1). Household crowding (≥3 persons per room) was slightly more frequent among infants with SARI-associated apnea compared to those without (32.9% vs. 28.1%; *p* = 0.499), though the difference was not statistically significant. In contrast, severe crowding (≥5 persons per room), a common characteristic of poor housing conditions, showed a clear association, being significantly more common in infants with apnea (20.3% vs. 11.1%; *p* = 0.042).

Beyond environmental exposures, several markers of biological vulnerability were strongly linked to apnea. Prematurity was strikingly more frequent among affected infants (49.3% vs. 13.2%; *p* < 0.001), both in moderate-to-late preterm (32–37 weeks; 40.3% vs. 11.7%; *p* < 0.001) and in very/extremely preterm infants (<32 weeks; 15.1% vs. 1.7%; *p* < 0.001). Consistently, infants with apnea had lower gestational age at birth (median 37 weeks [IQR 33–38] vs. 39 weeks [38–40]; *p* < 0.001), lower weight-for-age Z scores (median −2.38 [IQR −3.06 to −1.12] vs. −0.61 [−1.46 to 0.22]; *p* < 0.001), and higher rates of cesarean delivery (49.1% vs. 30.1%; *p* = 0.003).

Additional comorbidities were also more prevalent among infants with apnea, including intrauterine growth restriction (IUGR) (16.7% vs. 5.2%; *p* < 0.001), immunodeficiency (2.9% vs. 0.3%; *p* = 0.029), congenital heart disease (7.1% vs. 2.6%; *p* = 0.004), and neurological disorders (5.9% vs. 0.7%; *p* = 0.003). Prior hospitalizations were more frequent in the apnea group (84.4% vs. 71.0%; *p* = 0.009), with neonatal intensive care unit (NICU) admissions being particularly common (40.3% vs. 12.3%; *p* < 0.001).

We next assessed the clinical characteristics and outcomes associated with apnea (Table 1). Infants with apnea were significantly younger than those without (median age 1.5 months [IQR 0.8–2.8] vs. 2.2 months [IQR 1.4–3.3]; *p* < 0.001). Oxygen saturation at admission was similar between groups (median 90% [IQR 89–93] vs. 90% [88–96]; *p* = 0.430). However, infants with apnea experienced markedly worse outcomes, with higher rates of PICU admission (61.0% vs. 16.2%; *p* < 0.001) and in-hospital mortality (9.5% vs. 1.1%; *p* < 0.001), underscoring the strong association between apnea and critical illness.

We then explored the relationship between respiratory viral detection and apnea (Table 1). Interestingly, RSV infection was inversely associated with apnea (OR 0.42, 95% CI 0.24–0.70; *p* < 0.001), while no significant associations were observed for rhinovirus, human metapneumovirus, influenza, or viral coinfections. Given the clinical relevance of RSV in young infants, a subgroup analysis restricted to RSV-positive cases was performed (Supplementary Table S1). Among 1288 RSV-positive infants under 5 months, 26 (2.0%) presented apnea. Risk factors in this subgroup mirrored those in the overall cohort, with prematurity, lower gestational age, intrauterine growth restriction, lower weight-for-age Z score, cesarean delivery, chronic illness, and younger age at presentation all significantly associated with apnea. Notably, extremely preterm infants (<32 weeks) had a markedly elevated risk (OR 14.56, 95% CI 3.14–50.46), as did those with IUGR (OR 6.13, 95% CI 1.96–16.08). Sex and exclusive breastfeeding were significantly different between groups. For less frequent conditions such as immunodeficiency and neurological disorders, estimates were significant but imprecise due to small event numbers.

**Figure 1:**
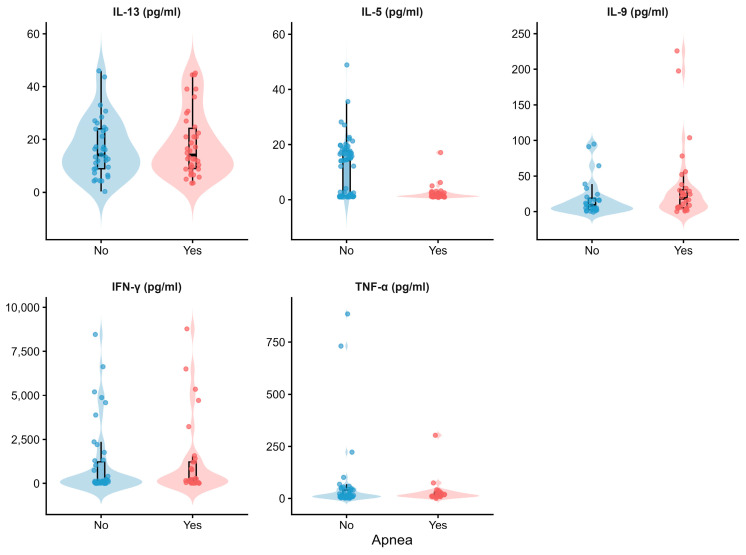
Distribution of nasal cytokine levels in infants with SARI according to apnea status. Violin plots show the distribution of cytokine concentrations (pg/mL) by apnea status (No/Yes). Boxplots inside each violin represent the median (center line) and interquartile range (IQR, box edges). Individual diamonds represent individual patient values. Y-axis scales were allowed to vary independently across panels to accommodate the different concentration ranges of each cytokine.

Finally, to investigate the role of airway inflammation in apnea episodes, cytokine levels in respiratory secretions were analyzed among infants with available biomarker data. IL-5 concentrations were significantly lower in infants with apnea compared to those without (median 1.39 pg/mL [IQR 1.14–2.30] vs. 14.47 pg/mL [1.33–17.77]; *p* < 0.001), and higher IL-5 levels were inversely associated with apnea in logistic regression (OR 0.23, 95% CI 0.12–0.44; *p* < 0.001). No significant differences were observed for IL-9, IL-13, IFN-γ, or TNFα (Figure 1). Infants with RSV infection showed higher IL-5 levels, but this pattern was absent among those with apnea (Figure S1a, *p* < 0.001). Across all groups, IL-5 concentrations were consistently lower in preterm infants (Figure S1b, *p* < 0.001).

### 3.2. Hierarchical Analysis of Factors Associated with Apnea

We then investigated independent factors using hierarchical multivariable logistic regression, incorporating variables identified as significant in the univariable analysis (Supplementary Table S2). The model was structured in three sequential levels, each reflecting a conceptual domain: sociodemographic (first level), biological (second level), and clinical/biomarkers (third level). Low birth weight, although associated with apnea in univariable analysis, was excluded due to collinearity with prematurity, indicating that its apparent effect was largely explained by gestational immaturity (Table 2).

At the first level, severe household crowding was significantly associated with apnea (OR 2.06, 95% CI 1.10–3.85; *p* = 0.023), suggesting that infants living in overcrowded conditions had approximately twice the odds of apnea compared to those in less crowded households.

At the second level, after adjustment for biological variables, severe crowding remained significant (OR 2.34, 95% CI 1.09–5.01; *p* = 0.029). Prematurity emerged as a strong predictor (OR 3.47, 95% CI 1.68–7.16; *p* = 0.001), alongside cesarean delivery (OR 2.00, 95% CI 1.06–3.75; *p* = 0.031) and prior NICU admission (OR 2.79, 95% CI 1.36–5.73; *p* = 0.005).

Finally, at the third level, analyses were restricted to infants with available biomarker data (n = 93). Although the reduced sample size widened confidence intervals, several associations remained robust. Cesarean section (OR 8.36, 95% CI 1.91–36.51; *p* = 0.005) and age under two months (OR 5.62, 95% CI 1.54–20.51; *p* = 0.009) were independently associated with apnea. RSV infection continued to show an inverse association (OR 0.21, 95% CI 0.05–0.93; *p* = 0.040). Importantly, IL-5 levels were inversely associated with apnea (OR 0.84, 95% CI 0.73–0.96; *p* = 0.011), indicating that higher IL-5 concentrations conferred reduced odds of apnea. Severe crowding, prematurity, and NICU admission did not reach statistical significance at this level, a finding best interpreted in the context of limited sample size rather than as evidence of diminished effect.

### 3.3. Severity Profile and Critical Outcomes Associated with Apnea

To further characterize the severity profile of apnea, we examined factors linked to critical illness among hospitalized infants, defined as those who either died during admission or required PICU care. Of the 422 infants meeting this definition, 47 (11.1%) had apnea. Within this critically ill subgroup, biological immaturity emerged as the dominant distinguishing feature (Table 3). Prematurity was nearly four times more frequent among infants with apnea compared to those without (48.8% vs. 14.7%; OR 5.51, 95% CI 2.68–11.35; *p* < 0.001), and lower gestational age was consistently associated with apnea in continuous analysis (OR 0.77 per week, 95% CI 0.70–0.84; *p* < 0.001). A history of NICU admission was also substantially more common in the apnea group (36.2% vs. 12.3%; OR 4.03, 95% CI 1.93–8.26; *p* < 0.001).

Consistent with findings in the overall cohort, RSV infection was less frequent among critically ill infants with apnea (37.5% vs. 67.0%; OR 0.29, 95% CI 0.15–0.57; *p* < 0.001). Moreover, IL-5 concentrations in respiratory secretions were markedly lower in infants with apnea (median 1.79 pg/mL [IQR 1.32–2.21] vs. 14.1 pg/mL [1.93–16.5]; OR 0.81, 95% CI 0.66–0.93; *p* = 0.008), reinforcing the inverse association between IL-5 levels and apnea risk.

## 4. Discussion

Apnea is one of the most severe complications of acute respiratory infection in early infancy, yet the biological, environmental, and virological factors that determine which infants are most at risk remain incompletely defined [3,6]. Using a hospital-based cohort of 2376 infants younger than 5 months admitted with SARI, we identified preterm birth, cesarean section, previous NICU admission, age under two months, and severe household crowding as independent predictors of apnea. RSV infection was inversely associated with apnea, and IL-5 levels in respiratory secretions were markedly lower in affected infants, pointing to a qualitatively distinct immune profile in this group. Together, these findings suggest that apnea during SARI in early infancy is driven primarily by biological immaturity and social vulnerability rather than by a specific respiratory pathogen. The confirmation of prematurity as a risk factor is consistent with previous evidence; the novel observations of this study are the independent associations of severe household crowding and of lower respiratory IL-5 concentrations with apnea.

Severe household crowding emerged as a significant and independent environmental risk factor for apnea, with its association persisting after sequential adjustment for prematurity and other biological and clinical variables. This suggests that its contribution operates through pathways distinct from, and likely additive to, biological immaturity. Crowding has previously been identified as an independent risk factor for community respiratory mortality in this population, and a proportion of those deaths may be attributable to unwitnessed apneic events occurring outside the hospital setting [22]. The present findings provide direct epidemiological support for that hypothesis. The biological mechanisms underlying this association remain unclear; crowding may increase exposure to respiratory pathogens and facilitate viral transmission [23,24] and likely co-occurs with other adverse social conditions, such as lack of sanitation, indoor air pollution, and limited healthcare access, that together may compound the risk of serious complications such as apnea [23].

Preterm birth also emerged as a strong and independent predictor of apnea, confirming its central role as a risk factor [4]. This association is biologically plausible, as brainstem respiratory centers demonstrate immature central and peripheral chemoreceptor responses and diminished neuromuscular control of upper airway patency. The degree of immaturity and severity of clinical symptoms are inversely correlated with gestational age [4]. Cesarean section emerged as one of the strongest independent risk factors for apnea in our cohort, even after adjustment for gestational age and other perinatal variables. This finding is consistent with prior evidence showing that infants born by cesarean section face significantly higher risks of adverse respiratory outcomes: in a previous study from our group, cesarean delivery was significantly associated with dying from RSV at home (OR 6.7; 95% CI 1.2–37.5), and apnea was identified as a plausible mechanism of death in several of these community fatalities, suggesting that impaired respiratory adaptation at birth may predispose vulnerable infants to life-threatening events outside the hospital setting [25]. From a biological standpoint, cesarean section, particularly when elective, deprives the infant of the hormonal and mechanical stimuli of labor that normally promote pulmonary fluid clearance and surfactant release, leading to impaired respiratory adaptation [26]. Altered breathing patterns and reduced respiratory drive have been documented in term infants delivered by cesarean section, and experimental models demonstrate that the mechanical stimuli of uterine contractions play a key role in initiating and sustaining neonatal respiratory behavior [27,28]. Furthermore, the absence of exposure to the maternal vaginal and gut microbiota during passage through the birth canal may alter the development of airway immune responses, potentially increasing reactivity during subsequent respiratory infections [29]. Taken together, these mechanisms may amplify the risk of apnea in biologically vulnerable infants, and cesarean delivery has also been identified as a risk factor for sudden infant death syndrome, a condition in which apnea is considered a potential terminal event [30]. Previous NICU admission likely captures residual biological vulnerability not fully accounted for by prematurity alone, as it was retained as an independent predictor even after adjustment for gestational age and other biological variables. Taken together, these findings point to biological immaturity as the dominant driver of apnea risk in this population, operating through multiple and partially overlapping pathways.

The inverse association between RSV infection and apnea observed in our study may appear counterintuitive, as RSV has traditionally been considered a major trigger of apnea in young infants [6,7,10]. Previous studies identified prematurity as the main predictor of apnea in RSV-positive infants, suggesting that the virus acts primarily as a precipitating factor in those already biologically predisposed [6,10]. The inverse association observed in our study is likely explained by the composition of the study population: since all infants were hospitalized with SARI, RSV-positive infants, who predominantly present with bronchiolitis, may represent a clinically distinct group from those who develop apnea. A direct comparison within the RSV-positive subgroup (Supplementary Table S1) supports this interpretation. Infants who developed apnea had substantially higher rates of prematurity (OR 5.20, 95% CI 2.24–11.53), extreme prematurity (OR 14.56, 95% CI 3.14–50.46), cesarean delivery (OR 3.30, 95% CI 1.32–8.62), intrauterine growth retardation (OR 6.13, 95% CI 1.96–16.08), and underlying chronic illness (OR 7.87, 95% CI 1.85–25.51), together with lower gestational age and lower weight-for-age at the time of the episode, than RSV-positive infants without apnea. Consistent with this interpretation, even within the RSV-positive subgroup, prematurity rather than viral etiology was the dominant predictor of apnea [6,10]. This phenotypic distinction is further supported by the cytokine data. IL-5 levels in respiratory secretions were significantly lower among infants with apnea in both the overall cohort and critically ill patients. IL-5 levels in respiratory secretions have previously been reported to be higher in RSV-infected infants compared to those infected by other pathogens [20], consistent with the predominant Th2 immune response characteristic of RSV-associated bronchiolitis. In this context, higher IL-5 may serve as a marker of the bronchiolitis phenotype rather than a direct modulator of apnea risk [31]. IL-5 is a canonical cytokine of Th2-polarized adaptive immunity; its consistent reduction in infants with apnea, independent of viral etiology, may therefore reflect a fundamentally more immature immunological state rather than a pathogen-specific effect. This interpretation aligns with the well-documented impairment of adaptive immune responses in preterm infants, who show reduced Th2 cytokine capacity alongside broadly diminished T-cell function [32]. IL-1β and PGE2, although mechanistically implicated in virus-associated apnea, could not be adequately evaluated in this cohort—IL-1β because it was measured in only a small number of samples, and PGE2 because it was not measured—and their direct assessment in respiratory secretions is an important direction for future work.

Our study has important limitations. Apnea diagnosis was based on parental descriptions of events at home or clinical observation by healthcare personnel during hospitalization, rather than objective methods such as polygraphy [33,34], which may have led to underdetection of subclinical episodes [3,33]. This should be considered when interpreting the magnitude of the reported associations. The biomarker subsample was substantially smaller than the full cohort, limiting statistical power and resulting in wider confidence intervals in the biomarker analyses. The association between lower IL-5 and apnea persisted after adjustment for prematurity but derives from a limited biomarker subset and should be regarded as exploratory and hypothesis-generating. Among infants with complete covariate data (n = 93), the events-per-variable ratio was approximately 5.6. Although below the traditionally cited threshold of 10, this is within the range supported as acceptable by simulation studies, particularly when predictors are pre-specified based on clinical and biological plausibility rather than data-driven selection [35] [Vittinghoff & McCulloch, 2007]. All covariates in our hierarchical model were pre-specified according to conceptual domain (sociodemographic, biological, immunological). A sensitivity analysis using Firth’s penalized logistic regression yielded consistent point estimates and direction of effect for RSV infection (OR 0.16, 95% CI 0.03–0.71) and IL-5 (OR 0.86, 95% CI 0.73–0.96), supporting the robustness of these two associations; estimates for other covariates showed wide confidence intervals, reflecting the limited sample size relative to the number of covariates jointly estimated; nonetheless, the direction of effect was consistent with the original model for all covariates. The RSV-positive subgroup analysis was similarly constrained by a small number of apnea events, limiting the power to detect associations in secondary analyses. Finally, a significant proportion of apnea-related deaths in this region likely occur outside healthcare facilities, which may have resulted in underestimation of apnea-associated mortality [36,37]. These limitations should be weighed against several important strengths. The population-based design, covering a well-defined birth cohort without private health insurance across 12 pediatric centers, minimizes the selection bias inherent in single-center or referral-based studies [14,38]. The hierarchical modeling strategy allowed for systematic disentangling of distal sociodemographic from proximal biological and clinical determinants, providing a more nuanced picture of the causal pathway to apnea. Viral detection by RT-PCR in all 2376 participants enabled rigorous assessment of pathogen-specific associations, an advantage over studies relying on clinical diagnosis alone. Finally, the simultaneous measurement of five cytokines in a subset of infants adds an immunological dimension that is largely absent from the existing literature on SARI-associated apnea.

## 5. Conclusions

In summary, this study identifies preterm birth, cesarean section, previous NICU admission, age under two months, and severe household crowding as independent predictors of apnea during acute respiratory illness in early infancy, while confirming the absence of a specific association between RSV infection and apnea. Biological immaturity and environmental adversity appear to be the main drivers of apnea risk in this setting, operating through mechanisms distinct from those driving RSV-related airway disease. In RSV-positive and critically ill infants, univariable analyses revealed consistent patterns, with prematurity, lower gestational age, and previous NICU admission dominating across all subgroups. The reduction in IL-5 levels in respiratory secretions of infants with apnea, observed in both the overall cohort and critically ill patients, is a novel finding that may reflect a more immature immune profile in this group, given that preterm infants characteristically show a Th2-skewed response and reduced adaptive immune capacity. Further research is needed to clarify the immunological mechanisms underlying apnea during acute respiratory infections and to inform targeted preventive strategies in vulnerable infant populations.

## Figures and Tables

**Table 1:** Characteristics of infants under 5 months of age included in the study, by presence or absence of apnea.

	Overall (%)	Apnea (%)	No Apnea (%)	*p*-Value
	N = 2324	N = 77	N = 2247	
Socio-demographic				
Teenage mother (<19 years old), n (%)	471 (20.60)	14 (20.59)	457 (21.63)	0.773
Low maternal educational level, n (%) *	295 (13.12)	10 (14.29)	285 (13.09)	0.720
Lack of sewage services, n (%)	1452 (62.37)	41 (61.19)	1411 (64.58)	0.095
Crowding, n (%)	(32.74)	18 (32.90)	647 (28.13)	0.499
Severe crowding (≥5 inhabitants/room), n (%)	232 (11.42)	13 (20.32)	219 (11.13)	0.042
Smoking at home, n (%)	1329 (61.97)	44 (61.97)	1285 (57.65)	0.542
Precarious housing, n (%)	512 (23.64)	16 (24.62)	496 (23.61)	0.770
Biological				
Male, n (%)	1319 (57.40)	44 (57.14)	1275 (57.41)	1.000
Prematurity, n (%)	326 (14.38)	36 (49.32)	290 (13.22)	<0.001
Moderate-to-late preterm (32–37 weeks’ gestation)	278 (12.53)	25 (40.32)	253 (11.73)	<0.001
Extremely preterm to very preterm (<32 weeks’ gestation)	48 (2.12)	11 (15.07)	37 (1.69)	<0.001
Cesarean section, n (%)	445 (30.88)	28 (49.12)	417 (30.13)	0.003
Intrauterine growth retardation, n (%)	119 (5.54)	11 (16.67)	108 (5.18)	<0.001
Gestational age in weeks, median (IQR)	39 (38 to 40)	37 (33 to 38)	39 (38 to 40)	<0.001
Weight Z Score at episode, median (IQR)	−0.64 (−1.51 to 0.2)	−2.38 (−3.06 to −1.12)	−0.61 (−1.46 to 0.22)	<0.001
Exclusive breastfeeding at the time of hospitalization, n (%)	334 (47.18)	14 (35.90)	320 (47.83)	0.150
Underlying chronic illness, n (%)	86 (3.80)	10 (3.47)	76 (14.49)	<0.001
Immunodeficiency, n (%)	9 (0.40)	2 (2.94)	7 (0.32)	0.029
Congenital heart disease, n (%)	60 (2.69)	5 (7.14)	55 (2.55)	0.004
Neurological disorder, n (%)	20 (0.89)	4 (5.88)	16 (0.73)	0.003
Previous hospitalizations, n (%)	1660 (71.43)	65 (84.42)	1595 (70.98)	0.009
Neonatal Intensive Care Unit, n (%)	307 (13.21)	31 (40.26)	276 (12.25)	<0.001
Clinical				
Age in months, median (IQR)	2.2 (1.4 to 3.2)	1.5 (0.8 to 2.8)	2.2 (1.4 to 3.3)	<0.001
Oxygen saturation at admission, median (IQR)	90 (89 to 93)	90 (89 to 93)	90 (88 to 96)	0.430
Pediatric intensive care unit admission (PICU), n (%)	412 (17.73)	47 (61.04)	365 (16.24)	<0.001
Death during hospitalization, n (%)	32 (1.41)	7 (9.46)	25 (1.14)	<0.001
RSV infection, n (%)	1288 (57.91)	26 (37.14)	1262 (58.59)	<0.001
RV infection, n (%)	370 (28.14)	11 (22.92)	359 (28.33)	0.420
hMPV infection, n (%)	129 (5.89)	3 (4.76)	126 (5.92)	0.790
Influenza infection, n (%)	40 (3.34)	2 (5.00)	36 (3.11)	0.365
Viral coinfection (≥2 respiratory viruses), n (%)	145 (2.07)	3 (3.90)	142 (6.32)	0.628
Biomarkers				
IL-5, median (IQR); n = 103	2.3 (1.25 to 16.36)	1.39 (1.14 to 2.30)	14.47 (1.33 to 17.77)	<0.001
IL-9, median (IQR) n = 58	11.86 (4.19 to 27.64)	18.40 (5.32 to 31.62)	9.44 (2.88 to 20.39)	0.136
IL-13, median (IQR); n = 73	14.16 (8.82 to 23.95)	14.12 (8.82 to 24.71)	14.16 (8.82 to 23.95)	0.813
IFN-γ, median (IQR); n = 71	137.27 (28.81 to 1292.17)	209.78 (41.28 to 1220.77)	106.5 (18.44 to 1292.17)	0.334
TNFα, median (IQR); n = 70	13.9 (8.00 to 38.00)	17.00 (9.60 to 30.33)	12.6 (7.00 to 40)	0.559

RSV: Respiratory syncytial virus; RV: rhinovirus; hMPV: human metapneumovirus; IL: interleukin. * Low maternal education level: mothers with incomplete primary education or less.

**Table 2:** Hierarchical multivariable logistic regression analysis of factors associated with apnea in children under 5 months of age. ORs with 95% CIs and *p*-values are presented for each stage of the model according to the predefined hierarchical framework.

	OR (95% CI)	*p*	OR (95% CI)	*p*	OR (95% CI)	*p*
Level 1						
Severe Crowding	2.06 (1.10 to 3.85)	0.023	2.34 (1.09 to 5.01)	0.029	4.38 (0.63 to 30.31)	0.134
Level 2						
Preterm			3.47 (1.68 to 7.16)	0.001	2.86 (0.42 to 19.58)	0.284
Cesarean section			2.00 (1.06 to 3.75)	0.031	8.36 (1.91 to 36.51)	0.005
Previous hospitalizations in NICU			2.79 (1.36 to 5.73)	0.005	0.59 (0.09 to 3.96)	0.586
Level 3						
Age in Months < 2					5.62 (1.54 to 20.51)	0.009
RSV infection					0.21 (0.047 to 0.93)	0.040
IL-5					0.84 (0.73 to 0.96)	0.011

NICU: Neonatal intensive care unit; RSV: respiratory syncytial virus; IL: interleukin. OR: odds ratio; CI: confidence interval; IL-5: interleukin-5.

**Table 3:** Characteristics of critically ill infants under 5 months of age by apnea status.

	Apnea N = 47	No Apnea N = 375	OR	*p*-Value
Severe crowding, n (%)	8 (23.5)	34 (11.3)	2.41 (0.87 to 6.05)	0.054
Intrauterine growth retardation, n (%)	6 (15.8)	35 (10.1)	1.66 (0.53 to 4.41)	0.270
Preterm < 37 weeks’ gestation, n (%)	21 (48.84)	53 (14.68)	5.51 (2.68 to 11.35)	<0.001
Age < 2 months, n (%)	33 (70.2)	251 (66.9)	1.16 (0.58 to 2.44)	0.740
Gestational age in weeks, median (IQR)	37 (34 to 38)	39 (38 to 40)	0.77 (0.70 to 0.84)	<0.001
Cesarean section, n (%)	14 (41.20)	90 (32.4)	1.46 (0.65 to 3.20)	0.340
Previous hospitalization in NICU, n (%)	17 (36.2)	46 (12.3)	4.03 (1.93 to 8.26)	<0.001
Congenital heart disease, n (%)	3 (7.3)	18 (5.2)	1.43 (0.26 to 5.23)	0.480
Neurological disease, n (%)	1 (2.6)	2 (0.6)	4.54 (0.08 to 89.22)	0.270
Need for mechanical ventilation, n (%)	11 (50)	20 (31.7)	2.11 (0.71 to 6.48)	0.200
RSV infection	15 (37.50)	238 (67.04)	0.29 (0.15 to 0.57)	<0.001
Length of stay in days, median (IQR)	9 (14 to 12)	9.5 (6 to 14)	0.96 (0.91 to 1.00)	0.131
IL-5, median (IQR)	1.79 (1.32 to 2.21)	14.1 (1.93 to 16.5)	0.81 (0.66 to 0.93)	0.008

NICU: neonatal intensive care unit; RSV: respiratory syncytial virus; IL: interleukin. Critically ill infants were defined as those who died during hospitalization or required admission to the pediatric intensive care unit. ORs, 95% CIs, and *p*-values are shown.

## Data Availability

The original contributions presented in this study are included in the article/Supplementary Materials. Further inquiries can be directed to the corresponding author.

## Supplementary Materials

The following supporting information can be downloaded at: https://www.mdpi.com/article/10.3390/v18070778/s1, Figure S1: IL-5 concentrations (pg/mL) according to apnea and RSV status (a) or prematurity (b); Table S1: Demographic and clinical characteristics of RSV-positive infants under 5 months of age, stratified by apnea status; Table S2: Univariable analysis. Forest plot of odds ratios for factors associated with apnea in infants younger than 5 months.
