# Supplementary material for: Biomarkers and Risk Factors Associated with Apnea in Hospitalized Infants with Acute Respiratory Infection
Source: Viruses. 2026 Jul 15;18(7):778. doi: 10.3390/v18070778 (PMC13431620; doi:10.3390/v18070778)
Supplement: Supplementary file 1 [file viruses-18-00778-s001.zip › viruses-4405385-supplementary.pdf]

Supplementary material

Biomarkers and Risk Factors Associated with Apnea in Hospitalized Infants with Acute Respiratory Infection

Julia Dvorkin [1,2,†], Maria Pico [1,†], Josefina L. Razzini [3], Romina Libster [4], Fernando P. Polack [4], Mauricio T. Caballero [1,2,*] and on behalf of The INFANT Respiratory Network [‡]

1 Centro Infant de Medicina Traslacional (CIMeT), Escuela de Bio y Nanotecnologías (EByN), Universidad Nacional de San Martín (UNSAM), General San Martín (ZIP 1650), Provincia de Buenos Aires, Argentina; jdvorkin@unsam.edu.ar (J.D.); mpico@iib.unsam.edu.ar (M.P.)
2 Consejo Nacional de Investigaciones Científicas y Técnicas (CONICET), Ciudad Autónoma de Buenos Aires (ZIP 1425), Argentina
3 Genetics, Vaccines and Pediatric Infectious Diseases Research Group (GENVIP), Instituto de Investigación Sanitaria de Santiago de Compostela (IDIS), University of Santiago de Compostela (USC), Galicia (ZIP 15706), Spain; josefinarazzini@gmail.com
4 Fundación INFANT, Ciudad Autónoma de Buenos Aires (ZIP 1406), Argentina; rlibster@infant.org.ar (R.L.); fpolack@infant.org.ar (F.P.P.)
* Correspondence: mcaballero@iib.unsam.edu.ar; Tel.:+54-1120331400 (ext. 6012)
† These authors contributed equally to this work.
‡ The INFANT Respiratory Network is listed in the Supplementary Materials.

The INFANT Respiratory Network: Luciano Alva Grimaldi (Hospital Zonal General de Agudos "Lucio Meléndez", Adrogué, Argentina), Andrea Sancilio (Hospital Interzonal General de Agudos "Evita", Lanús, Argentina), Karina Dueñas (Hospital Interzonal General de Agudos "Evita", Lanús, Argentina), Andrea Rodríguez (Hospital Zonal General de Agudos Descentralizado "Evita Pueblo," Berazategui, Argentina), Fernando Ferrero (Hospital General de Niños "Pedro de Elizalde," Buenos Aires, Argentina), Guadalupe Fernández Gago (Hospital Interzonal de Agudos Especializado en Pediatría "Sor María Ludovica", La Plata, Argentina).

Table S1. Demographic and clinical characteristics of RSV-positive infants under 5 months of age, stratified by apnea status.

	Overall (N = 1288)	Apnea (N = 26)	No apnea (N= 1262)	OR
Male, n (%)	724 (56.56)	15 (57.69)	709 (56.54)	1.05 (0.48 to 2.36)
Prematurity, n (%)	143 (11.30)	10 (38.46)	133 (10.73)	5.20 (2.24 to 11.53)
Moderate to late preterm (32-37 weeks' gestation), n (%)	129 (10.20)	7 (26.90)	122 (9.85)	3.37 (1.30 to 7.85)
Extremely preterm to very preterm (<32 weeks' gestation), n (%)	14 (1.11)	3 (11.54)	11 (0.89)	14.56 (3.14 to 50.46)
Caesarean section, n (%)	255 (30.04))	11 (57.89)	244 (29.40)	3.30 (1.32 to 8.62)
Intrauterine growth retardation, n (%)	56 (4.67)	5 (21.74)	51 (4.33)	6.13 (1.96 to 16.08)
Gestational age in weeks, median (IQR)	39 (38 to 40)	37 (35 to 38)	39 (38 to 40)	0.71 (0.63 to 0.80)
Weight Z Score at episode, median (IQR)	−0.56 (−1.46 to 0.21)	−2.45 (−2.95 to 1.63)	−0.56 (−1.39 to 0.23)	0.45 (0.33 to 0.60)

Exclusive breastfeeding at the time of hospitalization, n (%)	199 (48.77)	4 (36.36)	195 (49.12)	0.59 (0.15 to 1.99)
Age in months, mediana (IQR)	2.17 (1.35 to 3.25)	1.07(0.80 to 1.92)	2.20 (1.38 to 3.25)	0.45 (0.28 to 0.68)
Underlying chronic illnes, n (%)	33 (2.62%)	4 (16)	29 (2.32)	7.87 (1.85 to 25.51)
Inmunodeficiency, n (%)	5 (0.40)	1 (4.17)	4 (0.33)	13.32 (0.67 to 94.46)
Congenital heart disease, n (%)	24 (1.92)	3 (11.54)	21 (1.72)	7.47 (1.68 to 23.70)
Neurological disorder, n (%)	5 (0.40)	1 (4.17)	4 (0.33)	13.32 (0.67 to 94.46)

Odds ratios (ORs), 95% confidence intervals (95% CI), and p-values correspond to univariable logistic regression analyses within the RSV-positive subgroup.

Table S2. Univariable analysis. Forest plot of odds ratios for factors associated with apnea in infants younger than 5 months.

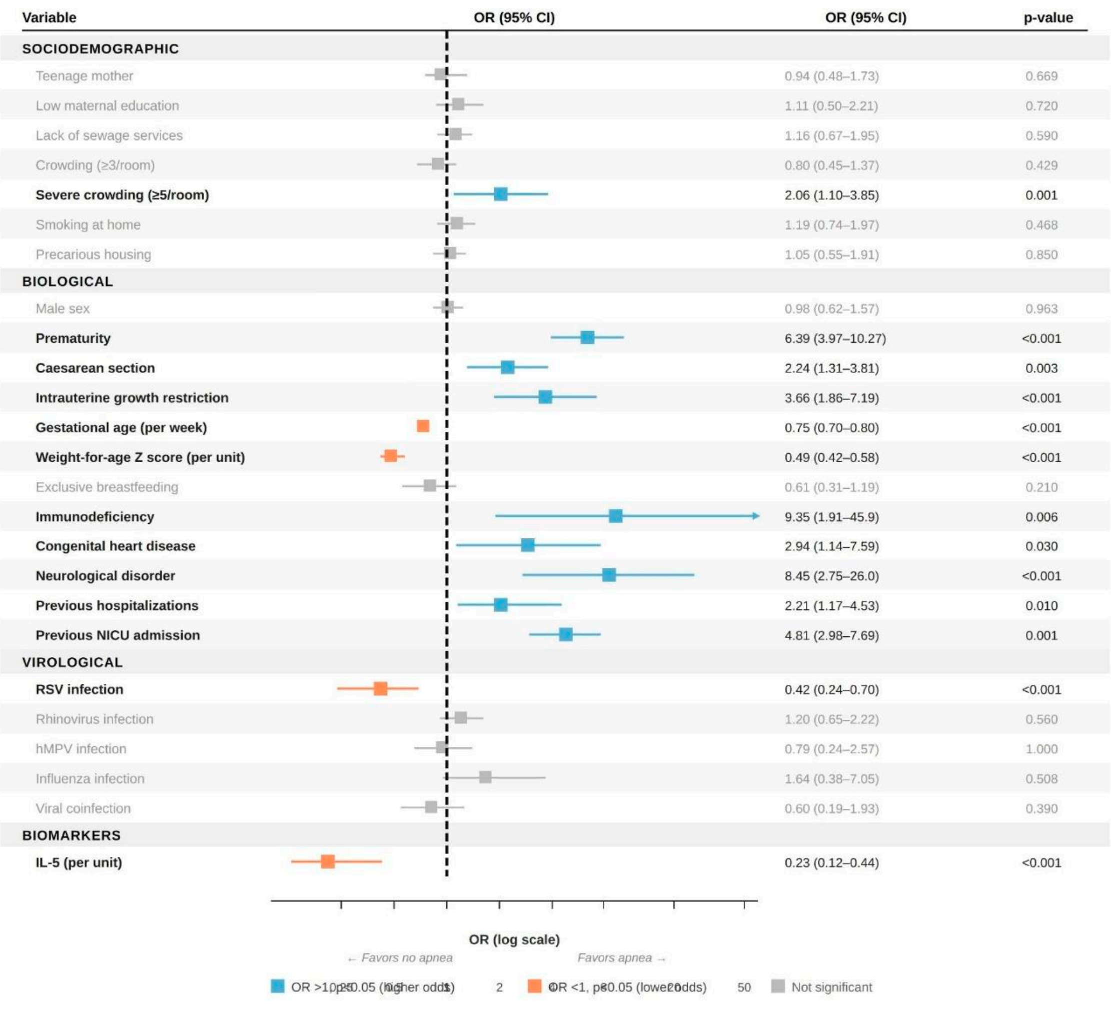

OR: odds ratio; CI: confidence interval; NICU: neonatal intensive care unit; RSV: respiratory syncytial virus; hMPV: human metapneumovirus; IUGR: intrauterine growth restriction. Bold labels and colored squares indicate statistically significant associations (p<0.05). Grey squares indicate non-significant associations. Dashed vertical line indicates OR=1.00. Arrow indicates CI extending beyond plot area.

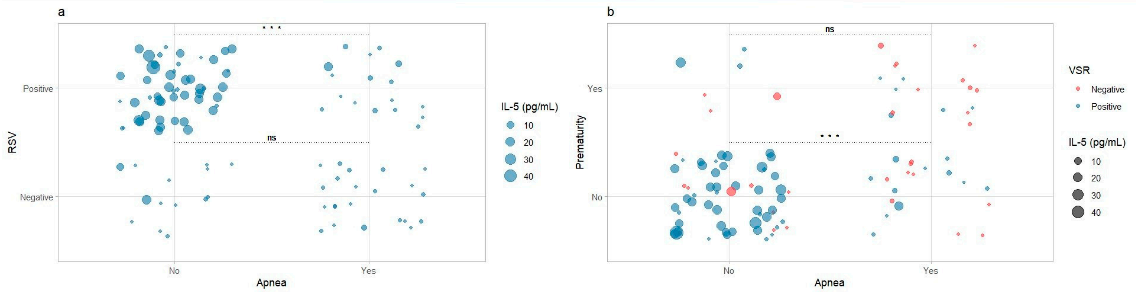

Figure S1. IL-5 concentrations (pg/mL) according to apnea and RSV status (a) or prematurity (b). Each bubble represents one patient; bubble size is proportional to IL-5 level. In panel (b), color indicates VSR status (blue: positive; red: negative). Brackets denote statistical comparisons (*** $p<0.001$; ns: not significant). Among RSV-positive infants, those without apnea showed significantly higher IL-5 levels compared to those with apnea; no significant difference was observed among RSV-negative infants. Similarly, among non-preterm infants, IL-5 levels were significantly higher in those without apnea, while no significant difference was found among preterm infants.
